# Interest of Pickering Emulsions for Sustainable Micro/Nanocellulose in Food and Cosmetic Applications

**DOI:** 10.3390/polym12102385

**Published:** 2020-10-16

**Authors:** Louise Perrin, Guillaume Gillet, Laurianne Gressin, Stephane Desobry

**Affiliations:** 1 Laboratoire d’Ingénierie des Biomolécules (LIBio), Université de Lorraine, 2 avenue de la Forêt de Haye, BP 20163-54505 Vandœuvre-lès-Nancy CEDEX, France; louise.perrin@univ-lorraine.fr; 2 SAS GENIALIS Route d’Achères, 18250 Henrichemont, France; g.gillet@genialis.fr (G.G.); l.gressin@genialis.fr (L.G.)

**Keywords:** pickering emulsion, unmodified cellulose, agricultural co-products, green processes, surfactant-free emulsion

## Abstract

In the present review, natural and non-toxic particles made of micro/nanocellulose were specifically targeted as stabilizers of emulsions located at dispersed and continuous phases interfaces (called Pickering Emulsions, PEs). PEs are biphasic systems stabilized by solid particles with a recent interest in food and cosmetic domains. PEs have been more and more studied in the last ten years due to their advantages compared to conventional emulsions with surfactants. PEs have already been stabilized with various types of particles and particularly cellulose. Even if some studies showed that PEs were more stable when cellulose was chemically modified, numerous other recent studies showed that unmodified micro/nanocellulose is also promising biomaterial to stabilize PEs. Micro/nanocelluloses can be extracted by various green processes from numerous agricultural wastes and co-products, as banana peels, corncob, ginkgo seed shells, lime residues, mangosteen rind, oil palm empty fruit bunches, pistachio shells, as well as wheat straw. Main green processes used to treat cellulose are grinding, high pressure homogenization, microfluidization, enzymatic hydrolysis, subcritical water, extrusion, electron beam irradiation, cryocrushing, microwaves or sonication. PEs formulated with cellulose clearly participate to a global sustainable development but, additional studies will be necessary to better understand PEs stability and improve properties.

## 1. Introduction

Emulsions are currently used in many fields, and especially in food, cosmetic and pharmaceutical industries. Emulsions are constituted of two, or more, immiscible phases, one is dispersed as droplets in the other. However, such systems are not thermodynamically stable and lead to phase separation to reduce interfacial surface between the two phases. The main way to stabilize these systems is to add amphiphilic molecules, called “surfactants”, which reduce interfacial energy and prevent phase separation [1,2]. These surfactants can cause adverse effects for health and environment, and these molecules present negative images for consumers looking for natural products [3,4,5,6]. Another way to stabilize emulsions is to use solid particles; this type of emulsions is called “Pickering Emulsions (PEs)”. The aim of the present review was to update knowledge about PEs and emphasize eco-friendly processes using cellulose to produce multiphasic systems.

### 1.1. Pickering Emulsions—General Aspects

Pickering emulsions (PEs) have been discovered more than a century ago [7,8], but research in this field really started in the 2000s, with around three thousand articles published in the last 20 years, according to the Web of Sciences Database. Among these numerous publications, some review articles have already been published on the PEs properties [9,10,11].

**Figure 1:**
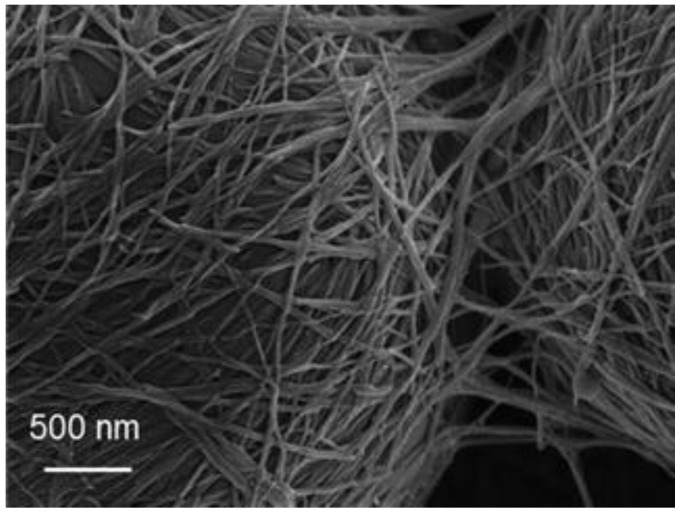
Scanning electron micrograph revealing physical barrier and network formation around and between droplets, by *Cladophora* cellulose nanocrystals (Reproduced from [15] with permission from the Royal Society of Chemistry).

PEs are stabilized by solid particles which can be of different morphologies, like fibrils, spheres, platelets, nanosheets, rods, cylinders, as well as cubes [11,12]. These particles have necessarily to be smaller than the droplets, and thus have generally a micro/nanoscale size. Many particles have already used to stabilize PEs like silica, calcium carbonate, graphene, polysaccharides (cellulose, chitosan, starch), proteins (from lentil, chickpea, lupin, soy), polyphenol crystals, or synthetic polymer particles [12,13,14]. Contrary to surfactants, these particles did not stabilize emulsions by reducing interfacial tension, but by forming a physical barrier and a network which constituted an obstacle between two contiguous droplets and avoided droplets coalescence (Figure 1).

**Figure 2:**
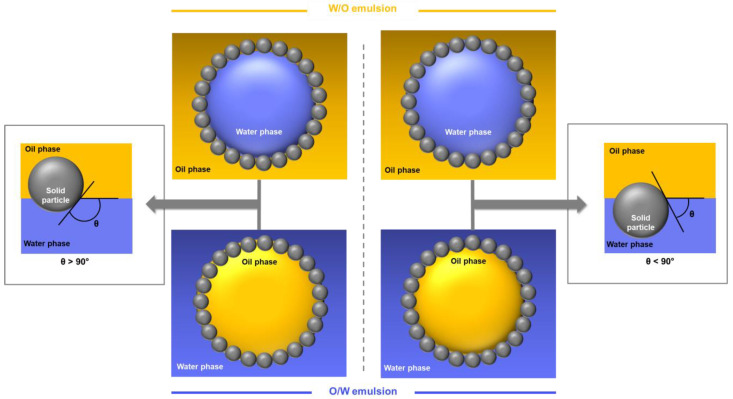
Schematic representations showing contact angle (θ) measured into water phase of spherical particles positioned at oil-water interface (left and right) and emulsion types corresponding, depending of volume fraction of each phase (middle).

Depending on particle nature, emulsions can be of type oil-in-water (O/W) or water-in-oil (W/O). More precisely, the emulsion type depends of particles wettability, which is determined by the contact angle in water (θ_w_), at oil-particle-water interface (Figure 2). Assuming that particles are perfect spheres, this angle is determined by the Young equation (Equation (1)):

(1) $$\cos(\theta_{w})=\frac{\gamma_{s-o}-\gamma_{s-w}}{\gamma_{o-w}}$$

where *γ_s-o_*, *γ_s-w_* and *γ_o-w_* represent the solid-oil, solid-water and oil-water interfacial tensions, respectively. If a particle has a contact angle below 90°, it is hydrophilic and, according to the Bancroft rule, enables to stabilize O/W emulsions. On the contrary, if the contact angle is above 90°, the particle is hydrophobic and enables to stabilize W/O emulsions [1,11,12,13]. Recently, authors studied also oil-in-oil emulsions stabilization by solid particles [16].

However, emulsion type also depends on the volume fraction of each phase. If the volume fraction of dispersed phase is close to 0.7, phase inversion happens. Thus, with particles having contact angle below 90°, when oil volume fraction increases up to 0.7, phase inversion from O/W to W/O can be observed. Likewise, with particles having contact angle above 90°, phase inversion from W/O to O/W can be obtained when water volume fraction increases up to 0.7 (Figure 2). However, phase inversions can only be obtained if emulsions are stable [17]. For example, phase inversions from O/W to W/O and from W/O to O/W could be observed with hydrophilic and hydrophobic silica particles, respectively [18], but with hydrophobized bacterial cellulose nanofibers, phase separation is obtained without phase inversion, when water volume fraction increased [19].

PEs present several advantages compared to conventional emulsions with surfactants and have been highly studied the last years. PEs would be more stable than conventional emulsions due to high energy adsorption of particles at oil-water interface. Indeed, particles adsorption and desorption energies depend of particle properties. More precisely, the closer θ is to 90° and the larger radius, the stronger the particle adsorption. Thus, according to these properties, particles adsorption energy at oil-water interface could lead to high adsorption energy at the interface, it is highly difficult to desorb them. Micro/nanoparticles could thus make it possible to obtain stable PEs for months [3,20]. However, additional research is still needed to obtain particles with optimal adsorption properties at oil-water interface, and to better understand PEs stability. Recent research progresses lead now to more and more stable PEs with natural and biodegradable particles, whereas most of surfactants are synthetic and not biodegradable in nature, causing toxicological and environmental problems [1,11,21].

### 1.2. Cellulose–General Aspects and Extraction

This review was focused on PEs stabilized with natural particles derived from cellulose, the most abundant natural polymer on earth. Cellulose is biodegradable, biocompatible, sustainable, renewable and inexpensive. Moreover, cellulose might be extracted from agricultural co-products and wastes. Use of these sources of cellulose to stabilize PEs does not compete with agricultural resources used for human consumption and can bring economical value from co-products [22,23]. All these advantages explain the important increase of research on PEs stabilized with cellulose, and some review articles have already been published [2,24,25] to show that cellulose microcrystals and microfibrils can stabilize PEs. Cellulose NanoCrystals (CNCs) and NanoFibrils (CNFs) demonstrated a better potential to stabilize PEs but nanocellulose production is relatively complex. Cellulose is composed of linear chains of β(1-4)-d-glucose units linked by O-glycosidic bonds. Several chains are stacked by strong hydrogen bonds constituting a rigid structure. Thus, to separate cellulose nanofibers, it has been often necessary to use hard treatments [26]. The main treatment used was acid hydrolysis, but this method presents several drawbacks, in particular large use of water, equipment corrosion, and most importantly, production of great amount of acid wastes [27,28,29]. Thus, even if cellulose is an eco-friendly polymer, production of nanocellulose may not be so eco-friendly. This is the reason why, new greener processes for nanocellulose production have been developed, like enzymatic hydrolysis, mechanical treatments as well as physical treatments (see Section 3.2. of this review).

Due to numerous surface hydroxyl groups, cellulose is a hydrophilic polymer and stabilizes O/W emulsions. However, several authors mention that it would be necessary to modify nanocellulose surface to obtain more stable PEs, because cellulose is too hydrophilic [23,30]. Thus, authors tried to modify chemically cellulose to improve PEs stability and properties, which makes cellulose even less eco-friendly.

In the context of agricultural wastes reduction, co-products valorization and sustainable development, this review is focused on examples of PEs stabilized with modified cellulose, and compared stability between PEs formulated with cellulose, both chemically unmodified and extracted with green processes from agricultural industry co-products.

## 2. Pickering Emulsions Stabilized with Modified Cellulose

Generally, chemical modifications of cellulose have been made by grafting or adsorbing molecules on pristine cellulose surface, such as hydroxypropylmethyl groups [31], sodium carboxymethyl cellulose [32], dodecylamine [33], polystyrene [34], cinnamoyl chloride and butyryl chloride [35], phenyltrimethoxysilane [36] or octenyl succinic anhydride [30,37]. Others molecules have been grafted on cellulose to bring specific properties at PEs, as *N*-isopropylacrylamide, a thermo-responsive molecule [38], or poly[2-(dimethylamino)ethyl methacrylate], a pH-responsive molecule [39]. These modifications were often made on nanocellulose, but some authors modified also microcellulose.

For instance, very recently, Ahsan et al. [32] modified microcrystalline cellulose by adsorbing sodium carboxymethyl cellulose (CMCNa) and studied the capacity of modified cellulose to stabilize PEs formulated with 1:9 dodecane/water ratio (*v*/*v*) and from 0.45 to 1.8% (*w*/*v*) modified cellulose. PEs stability depended on modified cellulose concentration, CMCNa molecular weight, pH and ionic strength. Despite a slight creaming, PEs were stable against coalescence, only if cellulose was modified by adsorbing CMCNa with high molecular weight. Moreover, pH from 3.0 to 11.0 and ionic strength from 0 to 100 mM of NaCl affected PEs stability. With 1.35% (*w*/*v*) cellulose, a creaming or a phase separation could be observed, depending on CMCNa molecular weight. These results showed the importance of the molecule grafted on cellulose on emulsion stability.

Another example was the CNCs modification with octenyl succinic anhydride (OSA), a molecule used to modify starch in food industry [30]. This modification increased CNCs hydrophobicity and improved their emulsifying capacity. PEs prepared with sunflower oil enriched in propionic and butyric acids, at 2:8 oil/water ratio (*w*/*w*) and 1.0% (*w*/*w*) modified CNCs, stored under refrigerated conditions, were stable for 4 weeks with creaming observed after two weeks. These PEs were also strongly resistant to coalescence for all pH (2.0–7.0) and ionic strengths (0–150 mM NaCl) tested by the authors. These PEs were sensitive to flocculation, when pH was below 4.0, or ionic strength above 20 mM NaCl. Moreover, a control with unmodified CNCs was made and a phase separation was observed immediately after emulsification. According to this article, CNCs modifications were necessary to obtain stable PEs.

These two first examples used simple and relatively green processes to modify cellulose, but grafted unnatural radicals on cellulose. Nevertheless, some methods to modify cellulose may be harmful for health and/or environment. Tang et al. [34,35] developed a strategy to produce amphiphilic nanocellulose (CNCs and CNFs) by grafting hydrophobic molecules via reductive amination. This modified nanocellulose was more effective than unmodified nanocellulose to stabilize PEs and would be biocompatible and environment friendly. However, the process used to modify nanocellulose required several solvents, as *N*,*N*-dimethylformamide, tetrahydrofuran and acetone, chemical molecules, as sodium cyanoborohydride, and hydrophobic molecule grafted on nanocellulose. This method to modify nanocellulose could lead solvent wastes and modified nanocellulose might also contain residual traces of solvents and chemical molecules, which would be unacceptable for many applications.

Even if modified cellulose did not totally avoid PEs stabilization, and particularly creaming, the three examples showed that modified cellulose use was more effective to stabilize PEs than pristine cellulose. However, numerous recent studies used unmodified cellulose to stabilize PEs. The next part of the present review is focused on PEs stabilized with unmodified cellulose, to study cellulose capacity to formulate green and eco-friendly PEs, or the necessity to modify cellulose to obtain stable PEs.

## 3. Eco-Friendly Pickering Emulsions

### 3.1. PEs Stabilized with Cellulose from Vegetal Co-Products

Cellulose is the main polymer constitutive of plant walls and can be extracted from numerous vegetal resources. To stabilize PEs, cellulose was essentially extracted from cotton and wood pulp [2], but recent researches were more and more interested by cellulose extracted from co-products (Table 1) to reduce and valorize agricultural wastes, without competing with human food and industrial activity. To stabilize PEs, this cellulose extracted from co-products was used in micro and nanocellulose form.

For example, in 2013, Winuprasith and Suphantharika used mangosteen rind, a waste produced by food and pharmaceutical industries, to produce Cellulose MicroFribrils (CMFs) and formulate PEs [41,42]. PEs formulated with 1:9 soybean oil/water ratio (*w*/*w*) and 0.45 or 0.63% (*w*/*w*) CMFs demonstrated high stability against coalescence and creaming. This stability was due to gel-like network formation, around droplets, increasing PEs viscosity, and reducing droplets mobility. Moreover, formulated with 3:7 soybean oil/water ratio (*w*/*w*), PEs were stable at different pH (3.0 to 8.0), ionic strengths (0 to 250 mM NaCl) and temperatures (30 to 90 °C). When CMFs concentration was superior to 0.10% (*w*/*w*), no creaming was observed under these surrounding conditions and demonstrated a weak creaming for PEs containing 0.10% (*w*/*w*) CMFs at pH 3.0. Thus, this study showed that unmodified microcellulose was a promising biomaterial to stabilize PEs, which would be even more stable than PEs stabilized with microcellulose modified with CMCNa [32]. Nevertheless, crystallinity index was lower for unmodified than modified microcellulose, but, in both cases, PEs were stabilized by barrier formation at oil-water interface and network formation between droplets by microcellulose.

Szlapak Franco et al. [50] extracted CNFs from peach palms (*Bactris gasipaes*) residues to stabilize PEs. Peach palm heart production generates high wastes amounts since almost 90% of trees are not used for human food. Thus, CNFs production from these residues contributes to sustainable development, through wastes reduction and farmers incomes increase. PEs formulated with 1:1 avocado oil/water ratio and 1.0% CNFs were stable, without creaming, for 30 days of storage at pH ranging from 3 to 11. However, PEs stability was slightly affected by temperature (80 °C). Moreover, PEs stabilized with CNFs were more stable than emulsions stabilized with 3.5% (*w*/*w*) sorbitan monostearate, a surfactant usually used in food, cosmetic and pharmaceutical industries. So, CNFs produced from peach palms would be able to replace some conventional surfactants.

Counter to Du Le et al. [30], Ni et al. [49] succeeded to stabilize PEs without modifying CNCs. CNCs were extracted from ginkgo seed shells, an agricultural waste usually burned or buried in the soil. PEs were formulated with different corn oil/water ratios (1:9 to 7:3 *v*/*v*) and CNCs concentrations (0.025% to 0.25% *w*/*v*). With 1:1 oil/water ratio (*v*/*v*) and CNCs concentration from 0.1 to 0.25% (*w*/*v*), stable PEs were obtained, without creaming for 3 months. Likewise, with 0.15% (*w*/*v*) CNCs, and oil/water ratios varying from 1:9 to 7:3 (*v*/*v*), no creaming was observed, but just an increasing of droplets size with increasing oil amount. PEs stability was tested under different surrounding conditions. PEs, formulated with 0.15% (*w*/*v*) CNCs, and 1:3 oil/water ratio (*v*/*v*) were stable under ionic strength below 100 mM. With 1:1 oil/water ratio (*v*/*v*), PEs were stable for temperatures varying from 20 to 80 °C. Thus, with weaker CNCs concentration and greater oil/water ratio, PEs were more stable than with modified CNCs with OSA [30]. Stability were explained by different morphology and ζ-potential between modified and unmodified CNCs. Unmodified CNCs were wider and longer than modified CNCs (25 nm vs. 2 to 4 nm wide, and 527 nm vs. 40 to 100 nm long, respectively), which might favor network formation between droplets, and so their immobility, preventing creaming and coalescence. Moreover, ζ-potential absolute value was higher for unmodified CNCs than modified CNCs (51.8 mV vs. 39.1 mV), which favored repulsion between droplets and avoided coalescence. Studies using carboxylated CNCs to stabilize PEs showed that a high ζ-potential improved repulsive forces between droplets and increased PEs stability, whereas low ζ-potential caused droplets flocculation or coalescence [26,40,48,51]. On the contrary, excess of charge density and high ζ-potential on CNCs surface had negative impact on PEs stability, due to repulsion between CNCs charges, causing insufficient density of CNCs at oil-water interface, and leading to coalescence and phase separation [52,53]. In these two studies, CNCs surface charge was essentially due to hydrolysis with sulfuric acid, causing esterification of sulfate anions and hydroxyl groups at CNCs surface. The differences in PEs stability between carboxylated and sulfated cellulose came from the presence of sulfate groups and not only from ζ-potential.

To avoid sulfate groups at cellulose surface, some authors used bacterial or plant cellulose hydrolyzed with milder conditions, with hydrochloric acid [22,54]. CNCs were successfully produced from agricultural wastes, pistachio shells, by hydrochloric acid treatment and presented better yield, and avoided cellulose crystalline regions damages and CNCs charge modifications. Moreover, promising PEs stability results were obtained with these CNCs. PEs, formulated with 1:9 corn oil/water ratio (*v*/*v*) and 1.5% (*w*/*w*) CNCs, had a stability index of 88.3% (calculated as the ratio between remaining emulsions volume after 28 days of storage and initial emulsion volume) [22]. However, Ni et al. [49] produced CNCs by sulfuric acid treatment and their PEs were more stable than PEs formulated with CNCs hydrolyzed by acid hydrochloric. Thus, roles of sulfate groups and ζ-potential on PEs stability need additional research.

These examples showed that cellulose extracted from agricultural co-products and unmodified could be promising biopolymer to stabilize eco-friendly and sustainable PEs. CNFs and CNCs have often been produced by acid hydrolysis, whose role on PEs stability has not been fully understood and this treatment caused undesirable acid wastes. Thus, new processes are emerging to produce CNCs and CNFs with greener methods.

### 3.2. Green Processes of Nanocellulose Preparation

Grinding is a green mechanical process to produce nanocellulose. Different grinders types can be used, as media mill [23], colloidal mill [50], or ball mill [55]. However, one of the grinding drawbacks is the size polydispersity of nanocellulose. For example, Lu et al. [23] grounded cellulose powders with a media mill, and CNFs sizes varying from 38 to 671 nm were obtained. However, this polydispersity did not avoid obtaining stable PEs, formulated with 1:1 oil/water ratio and 0.2 to 3.7% (*w*/*w*) CNFs, for 1 month of storage. PEs, formulated with 1.9% (*w*/*w*) CNFs, were stable under pH varying from 3.0 to 9.0, but creamed slightly under ionic strength (1 to 100 mM). These results showed that stable PEs could be obtained with nanocellulose extracted by green processes without acid hydrolysis.

High Pressure Homogenization (HPH) is another mechanical method used to produce CNFs and CNCs. In this method, cellulose passes through a small nozzle, with high pressure, causing cellulose cleavage at nanoscale [29]. Before to use HPH to produce nanocellulose, a pre-treatment, reducing cellulose size is often necessary, as grinding [46], hydrolysis with sulfuric acid [49], as well as ultra-sonication [56]. Nanocellulose produced by HPH might be used to stabilize PEs. For example, Li et al. [46] produced CNFs from *Miscanthus floridulus*, a fast-growing plant with limited industrial uses. *Miscanthus floridulus* straw was ground, delignified by alkali treatment and bleaching, and treated with HPH for 20, 30, 50 and 80 cycle times, at 150 MPa. Then, PEs were formulated with 1:9 dodecane/water ratio (*v*/*v*) and 0.20% (*w*/*w*) CNFs. The highest stability was obtained with CNFs homogenized during 30 cycle times with a creaming index below 8% after 14 days of storage.

Microfluidization, a similar process to HPH, could be used to produce nanocellulose from agricultural co-products, as *Cladophora glomerata*, an abundant green algae little exploited [57], oil palm mesocarp fibers, an industrial residue from palm oil production [58], hemp wastes [59], olive tree pruning residues [60], trunk of banana trees [61], as well as bagasse, a residual fibrous material from extraction of *Agave tequilana* juice [62]. However, microfluidization allowed generally to obtain long cellulose fibrils of several micrometers [59], so this process was often coupled with chemical pretreatments, as acid hydrolysis [58], TEMPO-oxidation [60], or organosolv process [62]. However, recently, greener technology was used, as high-shear homogenization [61].

Grinding, HPH and microfluidization are promising processes to produce nanocellulose, but present a major drawback, their great energy consumption. Enzymatic hydrolysis is another green method and less energy consuming used to generate CNFs or CNCs. The main enzymes used are cellulases, as endoglucanase and β-glucosidase, and xylanase [45,63,64]. For example, Costa et al. [45] used xylanase to produce CNFs from banana peels. Xylanase hydrolyzed all hemicelluloses and initiated hydrolysis of β(1-4) glycosidic bonds of cellulose amorphous regions. CNFs obtained were used to stabilize PEs, formulated with 1:9 sunflower oil/water ratio (*w*/*w*) and 0.01% (*w*/*w*) CNFs. PEs creamed after 1 day of storage, and phase separation was observed after 6 days. However, CNFs concentration used were very weak, so these results were encouraging, and stable PEs might be obtained with CNFs extracted by enzymatic hydrolysis from agricultural wastes, with higher CNFs concentrations.

Other green processes were promising to produce nanocellulose, as subcritical water, extrusion, electron beam irradiation, cryocrushing, microwave, as well as sonication. Subcritical water was used to produce CNFs. PEs, formulated with 3:7 almond oil/water ratio (*v*/*v*) and 1.0 to 5.0% (*w*/*w*) CNFs, were obtained with no phase separation, but creaming were observed after 24 h of storage [65]. Extrusion has the advantage to use cellulose slurry with high solid content, contrary to HPH or microfluidizer, which decreases production costs due to the fast procedure [66]. To decrease even more the energy consumption by limiting number of passes in extruder, this process could be combined with HPH and a pretreatment for cellulose defibrillation, as enzymatic hydrolysis with endoglucanase [67].

Electron beam irradiation could be also used to produce nanocellulose. For example, cellulose fibers from tall goldenrod (*Solidago altissima* L.), an invasive plant in Korea, were transformed by alkali treatment and bleaching, then by electron beam. When adsorbed dose increased from 50 to 300 kGy, cellulose fibrils width decreased from 10 µm to 160 nm [68]. Thus, to obtain nanocellulose, it was necessary to expose cellulose at high radiation doses, but, only doses inferior to 60 kGy would be safe for health [69]. Thus, this green process would be unacceptable for many applications, as food, cosmetic and pharmaceutical products. Cryocrushing consisted to crush cellulose immersed in liquid nitrogen. Ice crystals formed exert pressure, leading cell wall rupture and so, liberating cellulose fibrils. However, fibrils diameter was of the order of micrometers [70], thus this method was often combined with other green processes. For example, CNFs were extracted by cryocrushing associated with enzymatic hydrolysis and/or sonication, to valorize oil palm empty fruit bunches [71]. Microwaves have also been often combined with other treatments to produce nanocellulose. For instance, CNFs were extracted by steam explosion, microwave-assisted alkali treatment, and microfluidization, from wheat straw [72], or by microwave, high-shear homogenization and HPH, to valorize lime (*Citrus aurantifolia*) residues [73]. Likewise, sonication has also been associated with other processes. For instance, to valorize cassava (*Manihot esculenta*) peels, an agricultural waste, cellulose was isolated by alkali treatment, bleaching, and treated by sonication and high-shear homogenization to obtain CNFs [74].

All these green processes limited chemical reagents use and allowed valorizing agricultural wastes by producing eco-friendly nanocellulose. This nanocellulose might be then used in many fields, and particularly, like some authors have already done, as Pickering stabilizers, to formulate sustainable PEs. However, these green processes allowed essentially obtaining nanocellulose in the form of CNFs and no CNCs. However, that was not a major drawback, as these two types of nanocellulose stabilized PEs as mentioned in the Section 3.1, and CNFs would enable even better stabilize PEs than CNCs, due to better ability to form expanded and strong networks between droplets [75].

### 3.3. Pickering Emulsions Stabilized with Mixtures of Natural Nanoparticles

Even if nanocellulose could be produced by green processes, prior alkali treatment and bleaching are generally required, to eliminate lignins, hemicelluloses and pectins, and purify cellulose. Raw materials have been often treated with sodium hydroxide [22,43,46,48,50], but potassium hydroxide [45], or sodium carbonate coupled with active oxygen [56] might be also used. Bleaching has been frequently carried out with sodium chlorite and/or sodium hypochlorite [22,46,50,68], but these treatments caused toxic products release. Lignocellulosic materials could be also blanched with hydrogen peroxide, a greener alternative [41,42,43,56]. Another green alternative to eliminate lignin is micro-organisms inoculation. For example, *Marasmius* sp. was used to delignify oil palm empty fruit bunches. This fungus had high ligninolytic activity thanks to numerous ligninolytic enzymes production, as laccase and lignin peroxidase. However, this treatment lasted almost 28 days [71].

To limit pre-treatments and reduce chemical reagents use, authors stabilized PEs with unbleached lignocellulosic material. For example, bamboo shoots were delignified with alkali treatment, and then treated with HPH to obtain nanoparticles. PEs, formulated with 1:9 dodecane/water ratio (*v*/*v*) and 0.3% (*w*/*w*) nanoparticles, were stable without creaming for 30 days, under pH varying from 3.0 to 9.0 and for temperatures ranging from 4 °C to 50 °C [76]. However, bamboo shoots are food fibers and their use to stabilize PEs in cosmetic products could compete with resources used for human consumption.

Other vegetal materials, as ground coffee wastes [77], apple pomace, or oat bran [78] were also used to stabilize PEs without alkali treatment or bleaching. Ground coffee contains a lot of hemicellulose, but also lignin, protein and cellulose [79], apple pomace is rich in cellulose, pectin, lignin, hemicellulose and protein [80], and oat bran contains dietary fibers, as cellulose and lignin, but also starch and protein [81]. However, to obtain stable PEs, particles content was generally higher than with purified nanocellulose. For instance, stable PEs, formulated with 1:1 sunflower oil/water ratio, were obtained with a minimum of 8% ground coffee [77].

Powders composition had an impact on PEs stability. For instance, PEs, formulated with 1:1 oil/water ratio and 5% apple pomace or oat bran powders, showed different stability, depending on powder composition. Apple pomace provided a good stability, due to higher protein content and pectin presence forming networks between droplets. PEs stabilized with oat bran powder were resistant to coalescence due to higher starch content. These examples showed that agricultural co-products particles might stabilize PEs without chemical treatments. However, these powders could dye PEs, as the brown color caused by apple pomace, undesirable in cosmetic products [78].

## 4. Conclusion and Perspectives

PEs have been more and more studied during the last twenty years to replace unnatural and petrochemical surfactants by biosourced particles. PEs have possible applications in numerous domains, as food, cosmetic, pharmaceutical, biotechnological, as well as others industrial applications as additives in paints.

Micro/nanocellulose is a promising natural material to formulate PEs. The main mechanisms enabling emulsion stabilizing are high adsorption of particles at oil-water interface to form barriers and networks between droplets. These mechanisms are more and more described in the literature, but additional studies are still needed (i) to better understand cellulose surface charge role on PEs stability, (ii) to optimize particles properties (radius and contact angle, in particular), and (iii) to build stable particles adsorption at oil-water interface.

Agricultural and food industries produced each year large amount of wastes and co-products, rich in cellulose. In the context of sustainable development, reducing these wastes is an important issue. Thus, numerous green processes were developed to extract micro- and nanocellulose, and limited use of polluting chemical reagents. Then, to bring value to them, these cellulose derivatives could be used as Pickering stabilizers. Stable PEs could be obtained without chemically modify cellulose. Thus, eco-friendly cellulose is an encouraging biomaterial to formulate sustainable.

However, nanoparticles could cause health troubles, which could limit nanocellulose use in food and cosmetic products. Nanocellulose toxicity was evaluated and the results obtained were contradictory. Most studies suggested that nanocellulose presented a limited risk to human health, but, a few studies showed potential undesirable effects of nanocellulose [82]. Thus, even if numerous authors mentioned that PEs were less toxic than conventional emulsions with surfactants, additional research is necessary to evaluate toxicity of PEs formulated with nanocellulose.

## Figures and Tables

**Table 1:** Summary of scientific articles reporting cellulose use from agricultural co-products to stabilize Pickering emulsions.

Cellulose Source	Cellulose Type and (Width; Length) or (Diameter) (nm)	Particle Concentration	Hydrophobic Phase and (Hydrophobic:Hydrophilic Phase Ratio) ^a^	References
Pistachio shells	CNCs (68.8)	0.1–1.5% (*w*/*w*)	Corn oil (1:9) (*v*/*v*)	[22]
Kelp (*Laminaria japonica*)	CNFs (tens of nanometers; a few micrometers)	0.01–0.09% (*w*/*w*)	Sunflower oil (2:8) (*v*/*v*)S	[26]
*Cladophora rupestris*	CNCs (20; 4000)	0.15–0.5% (*w*/*v*)	Hexadecane (3:7)	[15]
Corncob	CNCs (363)	0.05–0.2% (*w*/*w*)	d-limonene (1:9) (*w*/*w*)	[40]
Mangosteen (*Garcinia mangostana* L.) rind	CMFs (DP: 711 to 1003)	0.045–0.63% (*w*/*w*)	Soybean oil (1:9; 3:7) (*w*/*w*)	[41,42]
Old spears and stems of asparagus (*Asparagus officinalis* L.)	CNCs (tens nanometers; 178.2)	0.7% (*w*/*w*)	Palm oil (3:7) (*v*/*v*)	[43]
Rice bran	CNCs (12.7; 327)	0–0.5% (*w*/*w*)	Rice bran oil (1:1) (*w*/*v*)	[44]
Banana (*Musa paradisiaca*) peels	CNFs (3.4; 1492 to 3493)	0.01% (*w*/*w*)	Sunflower oil (1:9) (*w*/*w*)	[45]
*Miscanthus floridulus*	CNFs (3 to 7; 33 to 49)	0.05–0.2% (*w*/*w*)	Dodecane (1:9) (*v*/*v*)	[46]
Bamboo pulp	Regenerated cellulose (DP: 756)	0.6% (*w*/*w*)	Caprylic/capric triglyceride (1:9; 2:8; 3:7; 4:6; 1:1) (*v*/*w*)	[47]
Oil palm empty fruit bunches	CNFs (4; few micrometers)	0.08–0.8%(*w*/*v*)	Dodecane (2:8) (*w*/*w*)	[48]
Ginkgo seed shells	CNCs (25.11; 527)	0.025 to 0.25% (*w*/*v*)	Corn oil (1:9; 3:7; 1:1; 7:3) (*v*/*v*)	[49]
Palm peach (*Bactris gasipaes*) agro-industrial residues	CNFs	1% (*w*/*w*)	Avocado oil (1:1)	[50]

DP: Degree of Polymerization; ^a^ [Hydrophobic:Hydrophilic phase Ratio] corresponds to ratio between hydrophobic and hydrophilic phases. For example, [1:9] correspond to 1 volume of hydrophobic phase for 9 volumes of hydrophilic phase.
